# Changes in CSF Surface Tension in Relation to Surfactant Proteins in Children with Intraventricular Hemorrhage

**DOI:** 10.3390/brainsci12111440

**Published:** 2022-10-26

**Authors:** Rieka M. Reger, Anton Meinicke, Wolfgang Härtig, Matthias Knüpfer, Ulrich Thome, Stefan Schob, Matthias Krause

**Affiliations:** 1 Department of Neurosurgery, University Hospital Leipzig, 04103 Leipzig, Germany; 2 Paul Flechsig Institute for Brain Research, Leipzig University, 04103 Leipzig, Germany; 3 Department of Neonatology, University Hospital Leipzig, 04103 Leipzig, Germany; 4 Department of Neuroradiology, University Hospital Halle, 06120 Halle (Saale), Germany

**Keywords:** cerebrospinal fluid, children, intraventricular hemorrhage, surface tension, surfactant proteins

## Abstract

The regulation of surface tension (ST) by surfactants plays an important role in the human respiratory system but is largely unexplored in brain homeostasis. The aim of this study was to evaluate changes in ST in relation to surfactant proteins (SPs) in children with intraventricular hemorrhage (IVH). CSF samples from 93 patients were analyzed for ST with a force tensiometer and SP-A-D and -G with ELISA assays. Patients belonged to six groups: (i) IVH before primary intervention (PI), (ii) IVH 4–28 days after PI, (iii) IVH 44–357 days after PI, (iv) hydrocephalus, (v) sepsis and (vi) controls. We found indirect correlations and significant differences in ST and SPs (all *p* < 0.001; except for SP-C, *p* = 0.007). Post hoc analyses showed significantly decreased ST in IVH patients before PI compared with patients with hydrocephalus, sepsis or controls (*p* < 0.001), but it increased in IVH patients over time. All SPs were significantly elevated when comparing IVH patients before PI with controls (all *p* < 0.001; except for SP-C, *p* = 0.003). Children suffering from IVH displayed an increase in SPs and a decrease in ST as coping mechanisms to preserve CSF flow. The increase in ST over time could serve as prognostic marker for the healing process.

## 1. Introduction

Surfactant proteins (SPs) account for 10% of the pulmonary surface active agent (surfactant) and play an important role in lowering surface tension (ST) by interacting with lipids and regulating the surfactant production and reuptake [1]. Hydrophilic surfactant proteins A (SP-A) and D (SP-D) are considered to be part of the innate immune system, binding pathogens and acting as opsonins that activate lung defense mechanisms [2,3,4]. Moreover, they regulate the reuptake of surfactant membranes [2,5,6]. SP-A is also described as a stabilizing and protecting factor of the surfactant layer [7]. 

Surfactant proteins B (SP-B) and C (SP-C) are structurally and functionally distinct from SP-A and SP-D. Both are characterized as rheologically active, highly hydrophobic proteins that further stabilize the complex pulmonary surfactant layer [8,9,10]. These surfactant proteins not only possess rheologic abilities themselves but also play an important role in the regulation of surfactant production and recycling [1]. 

The recently discovered surfactant protein G (SP-G) apparently has both rheological and immunological features [11,12,13]. Pulmonary surfactants and their physiological role in lowering the intra-alveolar ST, together with their importance for the pathogenesis of lung diseases such as respiratory distress syndrome (RDS), pulmonary fibrosis or idiopathic interstitial pneumonia, have been studied comprehensively [14,15]. However, the functional role of the cerebral surfactant and its changes in the cerebral spinal fluid (CSF) have only been investigated under selected conditions, mostly concerning the ventricular system [13,16,17]. 

Lungs of immaturely born babies lack the ability to sufficiently produce surfactants, which results in increased alveolar surface tension and frequently manifests as Infant (I) RDS [18]. Comparably, intraventricular hemorrhage (IVH) in preterm infants may also be the consequence of an insufficient surfactant layer at the CSF–ependymal interface, causing higher shear forces and eventually hemorrhage [17]. 

The occurrence of cerebral SPs follows a distinct developmental timeline. For example, SP-A and SP-C are abundant in the brain of rat embryos, while SP-B and SP-D only occur in adult rat brains [19]. Preterm infants have a higher risk of developing IVH, because cerebral autoregulation to prevent changes in critical blood flow is not fully developed yet; therefore, hypercarbia, hypoxia and hypoglycemia increase the risk of IVH by causing cerebral vasodilatation [20]. Enhanced surfactant production, and thus decreased ST after birth, might be a mechanism to lower shear stress at the especially vulnerable CSF–tissue interface and a reaction to previous hemorrhage. An earlier study by Krause et al. already showed elevated levels of SP-A, SP-C and SP-G in the CSF of preterm infants after IVH compared with controls and patients suffering from hydrocephalus (HC) [17]. To extend these data, the present study aimed to examine ST in relation to changes in SP concentrations in prematurely born children with IVH before and after intervention compared with infants suffering from HC or brain infection and with healthy controls. 

## 2. Materials and Methods

### 2.1. Patients

We analyzed 110 CSF samples (93 patients), which were categorized into six different groups:

(i) Group 1: IVH without/before primary intervention (N = 24);
(ii) Group 2: IVH 4–28 days after primary intervention (N = 14);
(iii) Group 3: IVH 44–357 days after primary intervention (N = 14);
(iv) Group 4: Hydrocephalus (N = 14);
(v) Group 5: Sepsis (N = 7);
(vi) Group 6: Control group (N = 37).

We examined 52 CSF specimens from 35 individual neonatal patients obtained at different time points after intraventricular hemorrhage in the due course of hydrocephalus treatment. The gestational age of the neonatal patients ranged from 24 to 37 weeks. 

Thus, we studied follow-up specimens of the same patients in the first 3 groups: 8 specimens of 4 patients were analyzed in group 1 and group 2, 8 specimens of 4 patients in groups 2 and 3, 9 specimens of 3 patients in all 3 groups and 6 specimens of 3 patients in groups 1 and 3. 

These three groups were analyzed to evaluate changes in ST and SP concentrations after intervention and at follow-up. Patients in IVH group 1 were aged 2–29 days; patients in IVH group 2 were 11–49 days old; group 3 consisted of patients being 59–389 days old. The distribution of age can be found in Table 1. In IVH group 1, the initial CSF was obtained when hydrocephalus and IVH became an indication for CSF drainage. IVH group 2 contained follow-up specimens of the babies that received an external drainage for continuous drainage or at the time of re-tapping/re-intervention. In IVH group 3, all specimens were obtained when a permanent CSF diversion was necessary (ETV or shunt). 

We also measured samples from hydrocephalus patients (HC; group 4) without IVH (N = 14), obtaining the CSF during the patient’s first shunt insertion. The children were between 2 days and 14 years old, with 12 of these 14 patients being younger than 1.5 years.

The 5th group (sepsis) comprised CSF samples from 7 patients, aged 2–16 days, who suffered from neonatal sepsis.

The control group (group 6) was a heterogeneous collection of specimens whose donors were between 2 days and 84 years old without a conclusive proof of neurological pathologies, cerebral inflammations or other CSF abnormalities. Considering ethical reasons, a control group only consisting of healthy neonatal patients was not available. Most newborns who have the indication for a CSF puncture present diseases of the brain and are thus subjected to changes in CSF composition. Patient data and surfactant protein concentrations in groups 4 and 6 (HC and controls) were previously published elsewhere and were only used for control and comparison reasons [16,17]. 

All patients or caregivers gave their written informed consent for the scientific use and analysis of CFS samples and clinical data.

This study was approved by the institutional review board of Medical Faculty of Leipzig University (Ethikkommission Universität Leipzig Az 330-13-18112013).

### 2.2. CSF Surface Tension

The measurement of the air–CSF surface tension was performed using a force tensiometer (K100; Krüss GmbH, Hamburg, Germany) with CSF samples of at least 0.2 mL at 22 °C. Using a rod-shaped probe that detected ST based on the Wilhelmy method allowed the use of small volumes as described by the manufacturer (Krüss GmbH, Hamburg, Germany). The direct measurement of interfacial tension between CSF and ependyma in vivo is impossible. Therefore, ST between CSF and air was determined as a surrogate parameter.

### 2.3. Quantification of Surfactant Proteins

Surfactant protein concentrations in CSF samples were quantified using commercially available enzyme-linked immunosorbent assays (ELISAs; USCN, Wuhan, China). The concentrations of SP-A (SEA890Hu), SP-B (SEB622Hu), SP-C (SEB623Hu), SP-D (SEB039Hu) and SP-G (SED755Hu) were measured according to the manufacturer’s manual using a microplate spectrometer (Spectramax M5; Molecular 206 Devices, LLC, San Jose, CA, USA; software: SoftMax Pro 5). Protein concentrations (ng/mL) were calculated by measuring the absorbance at a wavelength of 450 nm against standard series and antigen concentrations according to the manufacturer’s guidelines. Minimum detection limits were as follows: SP-A, 3000 pg/mL; SP-B, 1.56 ng/mL; SP-C, 0.312 ng/mL; SP-D, 6.25 ng/mL; SP-G, 0.156 ng/mL. It was possible to extrapolate lower measured values using the standard curve. 

The determination of the cell count, as well as lactate, glucose and total protein concentrations, in the CSF samples was part of the routine CSF laboratory and was included in our study to evaluate their relationship with ST and SP concentrations. 

### 2.4. Statistical Analysis

Statistical analyses were performed with the ANOVA test, post hoc tests (Tukey’s HSD test, Dunnett’s test), non-parametric Kruskal–Wallis test, Spearman rho test for correlations and graphics as box plots with SPSS version 22 (SPSS Inc., Chicago, IL, USA).

A *p*-value < 0.05 and a confidence interval > 0.95 were considered statistically significant.

## 3. Results

ST was measured for all 110 CSF samples. Due to very low specimen volumes, not all ELISAs could be performed for all CSF samples (SP-A, N = 58; SP-G, N = 106; SP-C, N = 79; SP-D, N = 58; SP-B, N = 57). Routine CSF examinations were not available for all parameters (cell count, N = 100; lactate, N = 92; glucose, N = 99; total protein concentration, N = 100; Supplemental Table S1). The clinical and laboratory parameters of the six investigated groups, including the ST values and levels of SP-A-D and SP-G, are presented in Table 1.

### 3.1. Correlation Analyses

The Spearman rho test indicated that all following parameters correlated significantly with each other across all groups: ST, SP-A, SP-B, SP-D, SP-G, cell count, glucose, lactate and total protein concentration (all *p* < 0.001). SP-C showed significant correlations with SP-G, glucose (all *p* < 0.001), ST (*p* = 0.001), cell count (*p* = 0.004), total protein concentration (*p* = 0.005), SP-D (*p* = 0.022), SP-A (*p* = 0.041) and SP-B (*p* = 0.049). It correlated positively with SP-A, SP-B, SP-D, SP-G, cell count, lactate and protein (r_s_ < 0.5) and negatively with ST and glucose (r_s_ > −0.5) (Table 2). 

ST presented strong negative correlations with SP-A, SP-B, lactate and total CSF protein (all r_s_ ≤ −0.5). ST also correlated negatively with SP-D, SP-G and cell count (all −0.5 < r_s_ ≤ −0.3). In addition, a positive correlation could be detected between ST and glucose (r_s_ = 0.521).

Moreover, SP-A, SP-B, SP-D, SP-G, cell count and total protein concentration showed strong positive correlations with each other (all r_s_ ≥ 0.5).

Lactate presented strong positive correlations with SP-A, SP-B, SP-D and total protein concentration (all r_s_ ≥ 0.5), while it showed only mild positive correlations with SP-G and cell count (0.3 ≤ r_s_ < 0.5). 

Glucose levels showed strong negative correlations with SP-A, SP-B, SP-D, SP-G, lactate, cell count and protein (all r_s_ ≤ −0.5), as well as a strong positive one with ST (r_s_ ≥ 0.5).

The control group presented the largest age difference. However, there were no significant correlations between age and ST nor between age and SPs. Only SP-A showed a strong positive correlation with age (*p* < 0.001, r_s_ = 0.559).

### 3.2. Variance Analyses and Non-Parametric Tests

The comparison among investigated groups by means of ANOVAs (Supplemental Table S2) revealed significant differences for the mean values of ST, SP-A, SP-B, SP-D, SP-G, lactate, glucose, total CSF protein and age (all *p* < 0.001), as well as SP-C (*p* = 0.007). No significant differences could be found between the groups for cell count (*p* = 0.051). 

Next, we performed non-parametric tests such as the Kruskal–Wallis test (Supplemental Table S3). These tests were in accordance with the ANOVA results, revealing significant differences between the investigated groups for all parameters, as mentioned above, as well as cell count (*p* < 0.001) and SP-C (*p* = 0.002). 

**Figure 1:**
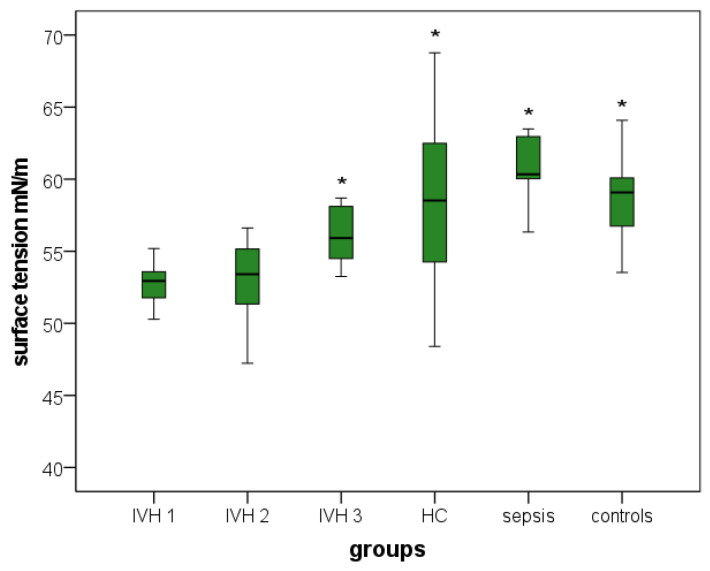
Boxplots: Distribution of surface tension values in the six investigated groups. IVH 1, patients with intraventricular hemorrhage without/before primary intervention; IVH 2, patients with intraventricular hemorrhage at 4–28 days; IVH 3, patients with intraventricular hemorrhage 44–357 days after primary intervention; HC, hydrocephalus patients between 2 days and 14 years old; sepsis, patients aged 2–16 days suffering from neonatal sepsis; controls, patients between 2 days and 84 years old without a conclusive proof of neurological pathologies, cerebral inflammations or other CSF abnormalities. Boxplots present results as medians between the 1st and 3rd quartiles. Whiskers comprise the range from minimum to maximum. Significant differences between groups in comparison with IVH group 1 are marked with an asterisk. * A *p*-value < 0.05 and a confidence interval > 0.95 were considered statistically significant.

**Figure 2:**
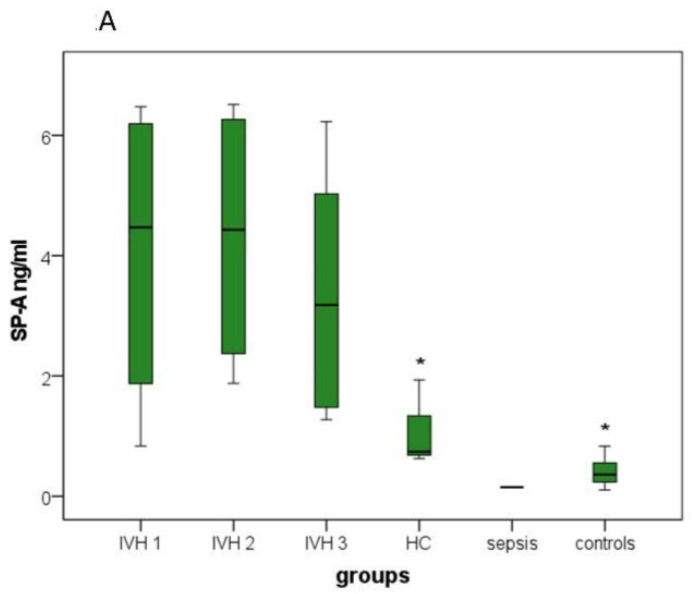

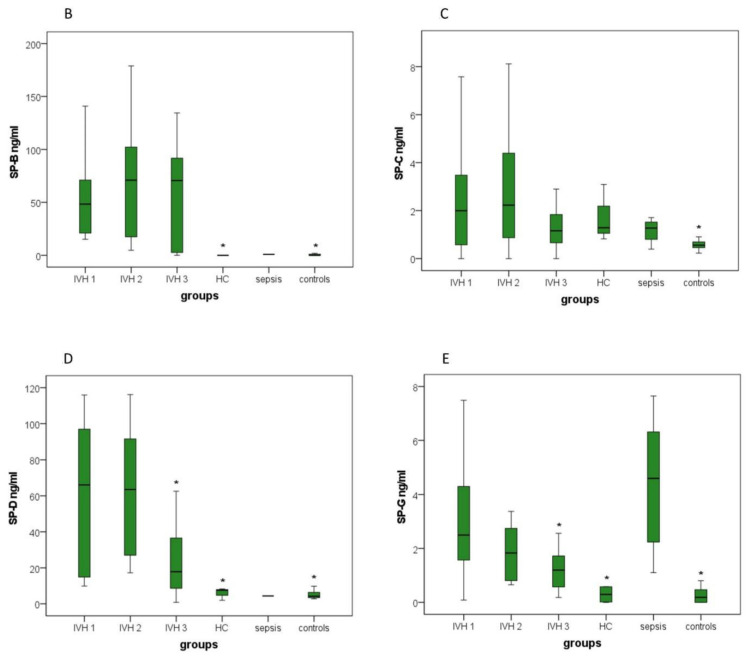
Boxplots: Distribution of surfactant protein levels in the six investigated groups: (**A**) SP-A levels, (**B**) SP-B levels; (**C**) SP-C levels, (**D**) SP-D levels and (**E**) SP-G levels. The sepsis group contained only one specimen in A, B and D. IVH group 1, patients with intraventricular hemorrhage without/before primary intervention; IVH 2, patients with intraventricular hemorrhage at 4–28 days; IVH 3, patients with intraventricular hemorrhage 44–357 days after primary intervention; HC, hydrocephalus patients between 2 days and 14 years old; sepsis, patients aged 2–16 days suffering from neonatal sepsis; controls, patients between 2 days and 84 years old without a conclusive proof of neurological pathologies, cerebral inflammations or other CSF abnormalities. Boxplots present results as medians between the 1st and 3rd quartiles. Whiskers comprise the range from minimum to maximum. Significant differences between groups in comparison with IVH group 1 are marked with an asterisk. * A *p*-value < 0.05 and a confidence interval > 0.95 were considered statistically significant.

**Figure 3:**
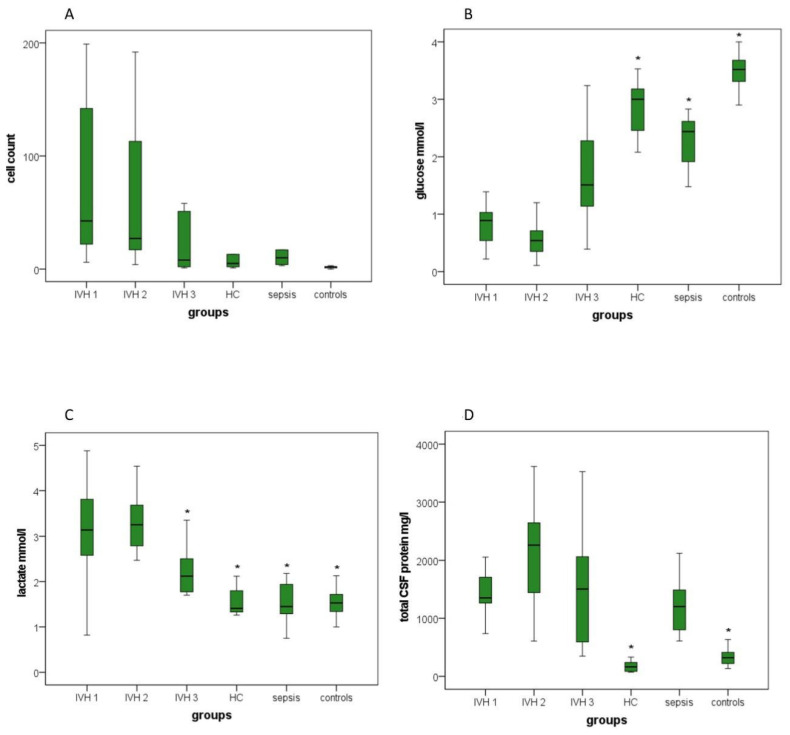
Boxplots: Distribution of cell count, glucose, lactate and total CSF protein concentrations in the six investigated groups: (**A**) cell count, (**B**) glucose concentration, (**C**) lactate concentration and (**D**) total CSF protein concentration. IVH group 1, patients with intraventricular hemorrhage without/before primary intervention; IVH2, patients with intraventricular hemorrhage at 4–28 days; IVH 3, patients with intraventricular hemorrhage 44–357 days after primary intervention; HC, hydrocephalus patients between 2 days and 14 years old; sepsis, patients aged 2–16 days suffering from neonatal sepsis; controls, patients between 2 days and 84 years old without a conclusive proof of neurological pathologies, cerebral inflammations or other CSF abnormalities. Boxplots present results as medians between the 1st and 3rd quartiles. Whiskers comprise the range from minimum to maximum. Significant differences between groups in comparison with IVH group 1 are marked with an asterisk. * A *p*-value < 0.05 and a confidence interval > 0.95 were considered statistically significant.

Boxplots demonstrate the distribution of all investigated parameters (Figure 1, Figure 2 and Figure 3).

Figure 1 shows the lowest ST values in the CSF of IVH group 1. The ST of IVH groups 2 and 3 exhibited somewhat increased ST values compared with IVH group 1. Notably, these values remained below the values of the three other groups (hydrocephalus, sepsis, controls). IVH group 3, and hydrocephalus, sepsis and control groups showed significantly higher ST values than IVH group 1.

Figure 2A displays significantly higher SP-A levels in IVH group 1 than in the hydrocephalus and control groups. Figure 2B shows significantly higher SP-B levels in IVH group 1 than in the hydrocephalus and control groups. Figure 2C displays significantly higher SP-C levels in IVH group 1 than in the control group. Figure 2D shows significantly higher SP-D levels in IVH group 1 than in IVH group 3, and hydrocephalus and control groups. Figure 2E reveals in patients of the sepsis group the highest SP-G protein levels, whereas patients in IVH group 1 possessed the second highest SP-G protein levels. The SP-G levels decreased from IVH group 1 to IVH group 2 and IVH group 3, to then the hydrocephalus group, showing a significant difference when comparing IVH group 1 with IVH group 3 and the hydrocephalus group. Additionally, a significant lower SP-G level was detected in the control group compared with IVH group 1.

Figure 3 displays the laboratory parameters for the study groups. No significant differences for cell count were detectable by comparing groups to IVH group 1, although the cell count was subsequently reduced from IVH group 1 to IVH group 2, to then IVH group 3. Compared with the control group, only the sepsis group exhibited a significantly increased cell count (Figure 3A). Glucose levels were increased in IVH group 3 compared with IVH group 1, but this difference was not significant. Significantly higher glucose levels were detected in the hydrocephalus, sepsis and control groups than in IVH group 1 (Figure 3B). Lactate levels were significantly decreased in IVH group 3 compared with IVH group 1. Significantly lower lactate levels were also detected in the hydrocephalus, sepsis and control groups than in IVH group 1 (Figure 3C). Significant lower levels of CSF protein were revealed in the hydrocephalus and control groups than in IVH group 1 (Figure 3D).

### 3.3. Post Hoc Analyses

#### 3.3.1. Surface Tension

The post hoc analyses using Tukey’s test showed that ST in IVH (group 1) was significantly decreased compared with group 3 (*p* = 0.009) and groups 4–6 (all *p* < 0.001) but not significantly different from that in group 2. A two-sided Dunnett’s test supported these findings, revealing significant differences between group 1 and groups 3–6 (group 3, *p* = 0.004; groups 4–6, all *p* < 0.001), but no differences were found when comparing groups 1 and 2 (Supplemental Table S4, Figure 1). 

#### 3.3.2. SP-A, SP-B and SP-D

Because of the low number of samples in the sepsis group (group 5), this one had to be excluded from the post hoc tests for SP-A, SP-B and SP-D.

Tukey’s test showed significant higher mean values for SP-A, SP-B and SP-D when comparing group 1 with groups 4 (SP-A, *p* = 0.017; SP-B, *p* = 0.047; SP-D, *p* = 0.005) and 6 (all *p* < 0.001). 

Dunnett’s test revealed equivalent results when comparing group 1 with group 4 (SP-A, *p* = 0.008; SP-B, *p* = 0.023; SP-D: *p* = 0.002) and group 6 (all *p* < 0.001). Moreover, SP-D in group 1 was significantly increased compared with that in group 3 in Tukey’s test (*p* = 0.027) and Dunnett’s test (*p* = 0.013; Supplemental Table S5, Figure 2A,B,D).

#### 3.3.3. SP-C

Tukey’s test revealed that the mean SP-C values of group 1 significantly exceed those of group 6 (*p* = 0.008). No significant differences could be found for the other groups compared to group 1. A two-sided Dunnett’s test confirmed the result (*p* = 0.003; Supplemental Table S4, Figure 2C). 

#### 3.3.4. SP-G

Comparable to the results for ST, Tukey’s test showed significant SP-G value differences when comparing group 1 with group 3 (*p* = 0.002) and with groups 4 and 6 (both *p* < 0.001), respectively. However, SP-G was significantly increased in group 1 in contrast to ST. There were no differences when comparing group 1 to groups 2 and 5.

A two-sided Dunnett’s test revealed the exact same SP-G value differences when comparing group 1 to groups 3, 4 and 6 and also no significant differences with respect to the other groups (Supplemental Table S4, Figure 2E).

#### 3.3.5. Cell Count

The post hoc analyses using Tukey’s and Dunnett’s tests did not reveal any significant differences between the mean values of cell count in groups 2–6 compared to group 1 (Supplemental Table S4). Compared with group 6, Tukey’s and Dunnett’s tests only showed a significantly increased cell count in group 5 (*p* = 0.03 and *p* = 0.012; Supplemental Table S4, Figure 3A).

#### 3.3.6. Lactate, Glucose and Total CSF Protein Concentration

Significant differences concerning the amount of lactate, glucose and total CSF protein concentration could be found when comparing group 1 to groups 4 and 6 (both *p* < 0.001) using Tukey’s test. Moreover, lactate and glucose concentrations differed significantly between groups 1 and 5 (*p* = 0.001). In addition, lactate showed a significant difference when comparing groups 1 and 3 (*p* = 0.028). 

Dunnett’s test confirmed the listed findings (Supplemental Table S4, Figure 3B–D). 

## 4. Discussion

The present study, to the best of our knowledge, is the first report about changes in surface tension in CSF samples from prematurely born infants suffering from IVH prior and post intervention. This is insofar novel and important, as changes in CSF composition have been reported, but the impact on the rheological properties remains largely unknown [13,16,17,21].

CSF surface tension was significantly reduced in preterm infants with IVH compared with controls and children with HC or sepsis. ST in children with IVH increased after intervention. Compared with pre-interventional samples, ST was only slightly increased shortly after intervention. However, children whose first intervention was more than 43 days prior, presented ST mean values closer to the ones with hydrocephalic conditions and controls than to those with untreated IVH. This may indicate that a decrease in ST represents a reaction to periventricular shear stress, a suspected mechanism of IVH development [17], while the normalization of ST over time after intervention may suggest a reparative process. By lowering the intracranial pressure and clearing the ventricular system, low ST might no longer be needed to prevent further shear stress, or the trigger for increased surfactant production is resolved. 

Pulmonary surfactants are known to be the main regulating element of ST in the lung [1]. Surfactant protein production and secretion depend on the impact of mechanical force on the alveolar epithelium [22]. Although cerebrospinal fluid ST is not as well investigated, former studies show the presence of SPs in CSF as well as in the lungs [16]. The significant indirect correlations among the surfactant proteins (SP-A, -B, -C, -D and -G) and ST may indicate that these proteins have an important regulatory effect on ST in CSF. This hypothesis is further corroborated by the existence of Kolmer–Agduhr interneurons, specified mechanosensory neurons that contact the CSF and project into regulatory nuclei, such as the hypothalamus [23].

Notably, the precise regulatory mechanisms of CSF surfactants have not yet been studied comprehensively. Therefore, interpretation in context of the well-investigated pulmonary system is required. The interaction of SP-A and SP-B is vital for surfactant secretion regulation. SP-A acts as an inhibitory signal for secretion, antagonizing SP-B, which induces secretion [24,25]. Moreover, SP-A induces the reuptake of surfactant by alveolar epithelial type II cells after utilized surfactant membranes are transformed into vesicles in a SP-D-dependent process [5,6]. SP-A and SP-G were proposed to modulate paravascular clearance after hemorrhage, promoting the phagocytosis of blood degradation products [17]. Comparably, blood in the ventricular system could be a trigger for increased surfactant turnover. The higher amount of these proteins in IVH than in HC might be explained by IVH-caused elevated intraventricular pressure combined with blood contamination. Comparing all investigated surfactant proteins, the strongest negative correlation appeared between ST and SP-A (r_s_ = −0.663), supporting our theory that elevated surfactant turnover is a reaction to the blood contamination of CSF. 

The elevated levels of lactate, total CSF protein and cell count in groups 1 and 2 were a consequence of blood contamination in the CSF of children with IVH, but these levels decreased over time, as seen in group 3. Furthermore, it also explained the reduced glucose concentrations in groups 1 and 2 compared with group 3, as glucose is consumed by red blood cells, which are abundant in the CSF of children suffering from IVH. The children of group 5, who suffered from brain infection, expectedly showed elevated total CSF protein, cell count and reduced glucose levels. Confirmed by our findings, Kratochvíl and Hrncír already stated the indirect correlations between total CSF protein concentration or cell count and ST [26]. The authors discussed, in accordance with data obtained by Brydon et al., that proteins are the main functional components of surface tension in CSF while not specifically studying SPs [26,27]. 

Nevertheless, as SPs are a distinct proportion of the overall protein concentration, these correlations are not surprising. Moreover, Brydon et al. reported that CSF had even lower ST values than pure protein solution at the same concentration [27]. This can be explained by the fact that SPs in CSF surfactants also interact with lipids [1]. In line with this, Kratochvíl and Hrncír assume that parts of the cell membranes that have surface-tension-lowering effects themselves become eventually part of the surfactant layer [26]. This assumption may explain the indirect correlation between cell count and ST. 

Künzel ascribed only minor rheological activities to the glucose concentration; therefore, they did not further study it [28]. Moreover, there are no other studies available investigating the connections between glucose and ST in CSF. We found a strong positive correlation between glucose and ST. However, elevated cell numbers were indirectly correlated to glucose levels, because cells metabolize glucose and release lactate. Therefore, cell count is the potential link among low glucose levels, elevated lactate levels and low ST.

These findings suggest that blood itself could have an impact on ST. However, as cell count did not show significant differences among the different groups, as indicated by ANOVAs and the respective post hoc tests, this is unlikely to explain the significant ST differences between the investigated groups.

Apart from that, we suggest that the measured surfactant proteins and their negative effect on ST do not predominantly relate to blood contamination itself. Two former studies support our theory: Künzel found a possible impact of blood contamination on ST in CSF but in a non-distinctive manner [28]. In their analysis concerning the dependence of blood ST on temperatures between 21 and 25 °C, which is comparable to our conditions, Rosina et al. detected mean values above 58 mN/m [29]. These mean ST values were higher than the mean values in our IVH group before intervention. Although the measurements cannot be compared directly to each other, these findings indicate that blood contamination alone could not have affected the surface tension to the extent of the values we measured. Moreover, previous studies of our work group demonstrated the CSF-borne production of surfactant proteins that reduce ST, as shown in this analysis [21]. In synopsis, we assume that the sole impact of blood contamination without active surfactant regulation processes does not justify our significant results. 

SP-G, as a more recently described member of the surfactant protein family, was found to possess a similar potential for the regulation of ST as SP-C and SP-B [11,12]. Comparable to our results regarding SP-B and SP-C, we also found a significant negative correlation between SP-G and ST, substantiating the initial study by Rausch et al. [11]. 

Additionally, SP-G seems to play an important role in host defense [13], which is supported by our findings of high SP-G levels in children with brain infection. The fact that SP-G concentrations were the highest, although ST also peaked in this group, prompts the interpretation that the role of SP-G in host defense is potentially superior to its function as a rheologically active protein. However, it certainly substantially contributes to the stabilization of the surfactant layer and the lowering of ST when no pathogens have to be eliminated, as shown in children suffering from IVH and HC.

Our study also confirmed significant differences in ST mean values accompanied by SP changes in the other investigated groups. Schob et al. already found elevated levels of SP-A and SP-C in patients suffering from acute HC or aqueductal stenosis compared with controls [16]. Our findings concentrated on the comparison between HC patients and IVH patients of group 1. HC patients (group 4) showed overall higher ST mean values than IVH patients before/without intervention (group 1), while SP-A, -B, -D and -G were significantly lower in HC patients than in group 1. This supports the theory that the attenuated level of ST in IVH patients might be explained by both, reactions to mechanical forces inside the ventricular system and surfactant regulatory mechanisms triggered by blood products resulting from hemorrhage. 

Children in the sepsis group showed significantly higher ST mean values than all other groups. We hypothesize that the surfactant proteins involved in host defense mechanisms are consumed during this activity. Acting as opsonins, SP-A and SP-D can bind directly to microbial cells [3]. SP-A protects the alveolar epithelium from damage by regulating the toxicity of immune cells towards the lung itself [30]. Moreover, SP-A, -C and -D are able to increase the permeability of microorganisms [1,31]. Extended analyses appear necessary, because previous studies solely focused on the immunological response in the lung and did not include patients with brain infection [1]. Furthermore, the elevated levels of SP-G during inflammation remain elusive, as the molecular mechanism of its immunological properties has not yet been elucidated. In addition, we studied a rather small sepsis group that should be enlarged in future investigations.

Due to ethical reasons, samples of healthy infants are rare. The heterogeneity of the control group was a limitation of our study. Age only directly correlated with SP-A in the control group. However, the significant elevation of SP-A in all IVH groups (Supplemental Table S5), which consisted of infants, compared with the control group suggests that the effects we found were intrinsic rather than age related, as previously described by Schob et al. [16].

## 5. Conclusions

ST in IVH patients before intervention was significantly decreased compared with patients with HC, sepsis or healthy controls but increased in IVH patients over time after intervention. In general, there was an indirect correlation between ST and surfactant proteins in all groups. All surfactant proteins were significantly elevated when comparing IVH patients before intervention with healthy controls. 

Our findings support the theory that an increase in CSF surfactant proteins could be an effect of excessive shear forces due to increased CSF pressure and might also be partly triggered by blood contamination. Therefore, the related ST decrease in the CSF of infants with IVH before primary intervention might be a coping mechanism to maintain CSF flow. The increase in ST over time after IVH could serve as a potential prognostic marker for the healing process and may reflect the resilience of patients. The correlations between surfactant proteins, glucose, lactose, cell count and total CSF protein, and ST, along with the increase in ST during the healing process, have to be investigated in further studies.

## Figures and Tables

**Table 1:** Overview of clinical factors and laboratory parameters for the six investigated groups.

	Group 1	Group 2	Group 3	Group 4	Group 5	Group 6
Group Description	IVH ^1^ without/before Intervention	IVH ^1^ 4–28 Days after Intervention	IVH ^1^ 44–357 Days after Intervention	Hydrocephalus	Sepsis and Brain Infection	Controls
N	24	14	14	14	7	37
Mean age (days)	3–29 (15.46)	11–49 (31.29)	59–389 (115.21)	2–5416 (637.92)	2–16 (7.57)	2–30,698 (12,093.81)
CSF surface tension (mN/m)	39.2–57.50 (52.06)	47.23–56.62 (52.87)	53.25–58.70 (56.23)	48.40–68.77 (58.32)	56.34–63.49 (60.88)	50.66–68.27 (58.55)
SP-A (ng/mL)	0.83–6.48 (3.87)	1.88–6.52 (4.32)	1.27–6.23 (3.39)	0.63–1.93 (1.10)	0.15 ^2^	0.11–0.83 (0.41)
SP-B (ng/mL)	15.14–176.06 (64.31)	4.75–178.92 (74.24)	0.00–134.54 (59.92)	0.00	0.92 ^2^	0.00–1.92 (0.47)
SP-C (ng/mL)	0.00–17.02 (3.17)	0.00–9.92 (3.28)	0.00–5.80 (1.60)	0.82–3.09 (1.73)	0.40–1.71 (1.16)	0.23–2.26 (0.65)
SP-D (ng/mL)	9.91–115.94 (59.18)	17.3–116.14 (63.18)	0.83–62.61 (24.07)	1.96–8.32 (5.96)	4.40 ^2^	2.81–9.85 (5.01)
SP-G (ng/mL)	0.09–9.38 (3.44)	0.65–8.42 (2.28)	0.18–2.56 (1.22)	0.00–4.06 (0.69)	1.11–7.65 (4.35)	0.00–2.79 (0.37)
Cell count	6–5729 (394.86)	4–724 (110.07)	1–551 (63.71)	1–68 (14.50)	3–7772 (1302.67)	0–58 (3.80)
Glucose (mmol/L)	0.22–4.34 (1.07)	0.11–1.28 (0.63)	0.39–3.24 (1.62)	2.08–3.53 (2.86)	1.48–2.83 (2.26)	2.59–4.84 (3.48)
Lactate (mmol/L)	0.82–4.88 (3.15)	1.40–4.54 (3.19)	1.70–3.65 (2.27)	1.26–2.12 (1.56)	0.75–2.18 (1.52)	1.00–4.90 (1.74)
Total CSF protein (mg/L)	383.30–5640.90 (1860.71)	607.60–3615.10 (2138.71)	350.40–3525.40 (1528.07)	73.00–864.80 (221.93)	610.00–2121.80 (1216.66)	137.00–4150.00 (492.62)

^1^ Intraventricular hemorrhage. ^2^ Only one sample. Values are given as ranges (means).

**Table 2:** Spearman rho test: correlation of clinical factors and parameters.

		SP-A ng/mL	SP-B ng/mL	SP-C ng/mL	SP-D ng/mL	SP-G ng/mL	Cell Count	Glucose	Lactose	Protein mg/L
ST mN/m	Corr. coeff.	−0.663	−0.577	−0.381	−0.450	−0.385	−0.476	0.521	−0.565	−0.504
	Sig. (2-side)	**<0.001**	**<0.001**	**0.001**	**<0.001**	**<0.001**	**<0.001**	**<0.001**	**<0.001**	**<0.001**
	N	58	57	79	58	106	100	99	92	100
SP-A ng/mL	Corr. coeff.		0.754	0.271	0.736	0.728	0.698	−0.605	0.662	0.743
	Sig. (2-sided)		**<0.001**	0.041	**<0.001**	**<0.001**	**<0.001**	**<0.001**	**<0.001**	**<0.001**
	N		57	57	58	57	50	49	48	51
SP-B ng/mL	Corr. coeff.			0.265	0.778	0.754	0.634	−0.644	0.520	0.788
	Sig. (2-sided)			0.049	**<0.001**	**<0.001**	**<0.001**	**<0.001**	**<0.001**	**<0.001**
	N			56	57	56	49	48	47	50
SP-C ng/mL	Corr. coeff.				0.302	0.424	0.341	−0.436	0.295	0.332
	Sig. (2-sided)				0.022	**<0.001**	**0.004**	**<0.001**	0.016	0.005
	N				57	78	71	69	66	71
SP-D ng/mL	Corr. coeff.					0.672	0.547	−00.659	0.521	0.748
	Sig. (2-sided)					**<0.001**	**<0.001**	**<0.001**	**<0.001**	**<0.001**
	N					57	50	49	48	51
SP-G ng/mL	Corr. coeff.						0.574	−0.607	0.489	0.738
	Sig. (2-sided)						**<0.001**	**<0.001**	**<0.001**	**<0.001**
	N						97	96	90	97
Cell Count	Corr. coeff.							−0.658	0.492	0.635
	Sig. (2-sided)							**<0.001**	**<0.001**	**<0.001**
	N							96	90	97
Glucose	Corr. coeff.								−0.669	−0.668
	Sig. (2-sided)								**<0.001**	**<0.001**
	N								90	96
Lactate	Corr. coeff.									0.634
	Sig. (2-sided)									**<0.001**
	N									89

Bonferroni test: alpha level of significance, *p* = 0.0045. All *p*-values ≤ 0.0045 are marked in bold face. Abbreviations: ST—surface tension; Corr. coeff.—correlation coefficient; Sig.—significance.

## Data Availability

All data are available in the manuscript and Supplementary Materials. Detailed datasets used and analyzed during the current study are available from the corresponding author upon reasonable request.

## Supplementary Materials

The following supporting information can be downloaded at: https://www.mdpi.com/article/10.3390/brainsci12111440/s1, Table S1: Overview of patients and probands with analyzed clinical factors and laboratory parameters, Table S2: ANOVA test, Table S3: Kruskal–Wallis test, Table S4: Post hoc tests—Tukey’s and Dunnett’s tests for ST, SP-G, SP-C, cell count, lactate, glucose and total CSF protein, Table S5: Post hoc tests—Tukey’s and Dunnett’s tests for SP-A, SP-B and SP-D, Table S6: Spearman rho test for correlations between age, and ST and SPs in the control group.
