# Supplementary material for: Changes in CSF Surface Tension in Relation to Surfactant Proteins in Children with Intraventricular Hemorrhage
Source: Brain Sci. 2022 Oct 26;12(11):1440. doi: 10.3390/brainsci12111440 (PMC9688901; doi:10.3390/brainsci12111440)
Supplement: Supplementary file 1 [file brainsci-12-01440-s001.zip › brainsci-1960988-supplementary.pdf]

Supplemental Tables

Changes in CSF Surface Tension in Relation to Surfactant Proteins in Children with Intraventricular Hemorrhage

Rieka M. Reger [1], Anton Meinicke [2], Wolfgang Härtig [2], Matthias Knüpfer [3], Ulrich Thome [3], Stefan Schob [4] and Matthias Krause [1,*]

Table S1: Overview of patients and probands with analyzed clinical factors and laboratory parameters

N	group 1	group 2	group 3	group 4	group 5	group 6	total
CSF Surface Tension	24	14	14	14	7	37	110
SP-A	10	6	6	3	1	32	58
SP-B	10	6	5	3	1	32	57
SP-C	18	10	11	3	4	33	79
SP-D	10	6	6	3	1	32	58
SP-G	22	13	14	14	7	36	106
Cell Count	22	14	14	14	6	30	100
Glucose	22	14	13	14	7	29	99
Lactate	21	12	12	13	5	29	92
Total CSF Protein	23	14	13	12	7	31	100
Age	24	14	14	14	7	37	110

Table S2: ANOVA test

Between groups 1, 2, 3, 4, 5, 6	Significance
ST	<0.001
SP-A	<0.001
SP-B	<0.001
SP-C	0.007
SP-D	<0.001
SP-G	<0.001
CC	0.051
Lac	<0.001
Glu	<0.001
Prot mg/l	<0.001

Abbreviations: ST: surface tension; CC: cell count; Lac: lactate; Glu: glucose; Prot: total CSF protein

Table S3: Kruskall-Wallis test

Between groups 1, 2, 3, 4, 5, 6	Significance
ST	<0.001
SP-A	<0.001
SP-B	<0.001
SP-C	0.002
SP-D	<0.001
SP-G	<0.001
CC	<0.001
Lac	<0.001
Glu	<0.001
Prot mg/l	<0.001

Abbreviations: ST: surface tension; CC: cell count; Lac: lactate; Glu: glucose; Prot: total CSF protein

Table S4: Post-hoc-tests - Tukey and Dunnett test for ST, SP-G, SP-C, cell count, lactate, glucose, total CSF protein

Multiple comparison

Dependent Variable		(I) Group_no.	(J) Group_no.	Significance
ST	Tukey-HSD	1	2	0.984
			3	0.009
			4	<0.001
			5	<0.001
			6	<0.001
	Dunnett-T (2-sided)[b]	2	1	0.952
		3	1	0.004
		4	1	<0.001
		5	1	<0.001
		6	1	<0.001
SP-G	Tukey-HSD	1	2	0.339
			3	0.002
			4	<0.001
			5	0.793
			6	<0.001
	Dunnett-T (2-sided)[b]	2	1	0.182
		3	1	<0.001
		4	1	<0.001
		5	1	0.607
		6	1	<0.001
SP-C	Tukey-HSD	1	2	1.00
			3	0.527
			4	0.929
			5	0.657
			6	0.008
	Dunnett-T (2-sided)[b]	2	1	1.00
		3	1	0.339
		4	1	0.839
		5	1	0.465
		6	1	0.003
CC	Tukey-HSD	1	2	0.949
			3	0.906
			4	0.843
			5	0.296
			6	0.675
			1	0.675

		6	2	0.999
			3	1.000
			4	1.000
			5	0.030
	Dunnett-T (2-sided)b	2	1	0.868
		3	1	0.785
		4	1	0.681
		5	1	0.155
		6	1	0.469
		1	6	0.489
		2	6	0.998
		3	6	1.000
		4	6	1.000
		5	6	0.012
Lac	Tukey-HSD	1	2	1.00
			3	0.028
			4	<0.001
			5	<0.001
			6	<0.001
	Dunnett-T (2-sided)b	2	1	1.00
		3	1	0.011
		4	1	<0.001
		5	1	<0.001
		6	1	<0.001
Glu	Tukey-HSD	1	2	0.371
			3	0.158
			4	<0.001
			5	<0.001
			6	<0.001
	Dunnett-T (2-sided)b	2	1	0.206
		3	1	0.074
		4	1	<0.001
		5	1	<0.001
		6	1	<0.001
Prot	Tukey-HSD	1	2	0.937
			3	0.884
			4	<0.001
			5	0.538
			6	<0.001
	Dunnett-T (2-sided)b	2	1	0.847
		3	1	0.751
		4	1	<0.001
		5	1	0.339
		6	1	<0.001

Age	Tukey-HSD	1	2	1.00
			3	1.00
			4	0.999
			5	1.00
			6	<0.001
		6	1	<0.001
			2	<0.001
			3	<0.001
			4	<0.001
			5	<0.001
	Dunnett-T (2-sided)[b]	2	1	1.00
		3	1	1.00
		4	1	0.998
		5	1	1.00
		6	1	<0.001

Abbreviations: ST: surface tension; CC: cell count; Lac: lactate; Glu: glucose; Prot: total CSF protein

Table S5: Post-hoc-tests - Tukey and Dunnett test for SP-A, SP-B, SP-D

Dependent Variable		(I) group-no.	(J) group-no.	Significance
SP-D ng/ml	Tukey-HSD	1	2	0.997
			3	0.027
			4	0.005
			6	<0.001
	Dunnett-T (2-sided)[b]	2	1	0.991
		3	1	0.013
		4	1	0.002
		6	1	<0.001
SP-B ng/ml	Tukey-HSD	1	2	0.980
			3	0.999
			4	0.047
			6	<0.001
	Dunnett-T (2-sided)[b]	2	1	0.953
		3	1	0.998
		4	1	0.023
		6	1	<0.001
SP-A ng/ml	Tukey-HSD	1	2	0.964
			3	0.953
			4	0.017
			6	<0.001
	Dunnett-T (2-sided)[b]	2	1	0.918
		3	1	0.898
		4	1	0.008
		6	1	<0.001

Abbreviations: ST: surface tension; CC: cell count; Lac: lactate; Glu: glucose; Prot: total CSF protein

Table S6: Spearman-Rho test for correlations of age with ST and SPs in the control group

Correlations

			Age	ST	SP-G	SP-D ng/ml	SP-C ng/ml	SP-B ng/ml	SP-A ng/ml
Spearman-Rho	Age	correlation coefficient	1.000	-0.168	-0.122	0.276	-0.274	0.195	0.559**
		Significance (2-sided)	.	0.320	0.480	0.126	0.123	0.284	<.001
		N	37	37	36	32	33	32	32

** significance <0.001

Abbreviations: ST: surface tension; CC: cell count; Lac: lactate; Glu: glucose; Prot: total CSF protein
